# First-line disease modifying treatments in pediatric-onset multiple sclerosis in Greece: therapy initiation at more advanced age is the main cause of treatment failure, in a retrospective observational study, with a cohort from a single Multiple Sclerosis Center

**DOI:** 10.1007/s10072-022-06431-y

**Published:** 2022-10-05

**Authors:** Charalampos Skarlis, Nikolaos Markoglou, Maria Gontika, Anastasia Bougea, Serafeim Katsavos, Artemios Artemiadis, George Chrousos, Marinos Dalakas, Leonidas Stefanis, Maria Anagnostouli

**Affiliations:** 1 Research Immunogenetics Laboratory, 1st Department of Neurology, Medical School, National and Kapodistrian University of Athens, Aeginition University Hospital, Athens, Greece; 2 1st Department of Neurology, Medical School, National and Kapodistrian University of Athens, NKUA, Aeginition University Hospital, Vassilisis Sofias Ave 72-74, 11528 Athens, Greece; 3 Aghia Sophia Children’s Hospital, University Research Institute of Maternal and Child Health and Precision Medicine and UNESCO Chair On Adolescent Health Care, National and Kapodistrian University of Athens, 11527 Athens, Greece; 4 Neuroimmunology Unit, Department of Pathophysiology, National and Kapodistrian University of Athens, Athens, Greece; 5 Department of Neurology, Thomas Jefferson University, Philadelphia, PA USA; 6 Multiple Sclerosis and Demyelinating Diseases Unit, 1st, Department of Neurology, Medical School, National and Kapodistrian University of Athens, Aeginition University Hospital, Athens, Greece

**Keywords:** Pediatric-onset multiple sclerosis (POMS), 1st line DMTs, Injectables, Oral, HLA, Outcome

## Abstract

**Objectives:**

Long-term immunomodulatory therapy of pediatric onset-multiple sclerosis (POMS) is based mainly on published case series and internationally agreed guidelines. Relevant studies in the Greek population are absent from the literature. The purpose of this study is to present data on the efficacy and safety of the 1st line immunomodulatory drugs in the treatment of POMS patients.

**Materials and methods:**

The present study included 27 patients meeting the IPMSSG criteria for POMS and who are monitored at the outpatient clinic of the Multiple Sclerosis and Demyelinating Diseases Unit (MSDDU), of the 1st Neurological Department, University Hospital of Aeginition. All patients received 1st line immunomodulatory drugs as initial therapy. Clinical, laboratory, and imaging parameters of the disease were recorded before and after treatment.

**Results:**

Post-treatment, a significant reduction of the relapse number (mean ± SD: 2.0 ± 1.0 vs 1.2 ± 1.6, *p* = 0.002), EDSS progression (mean ± SD: 1.5 ± 0.8 vs 0.9 ± 0.7, *p* = 0.005) and ARR (mean ± SD: 1.5 ± 0.7 vs 0.4 ± 0.5, *p* = 0.0001) was observed, while no changes were observed in the EDSS score, (mean ± SD: 1.8 ± 0.6 vs 1.9. 0.6, *p* = 0.60). Advanced age at treatment initiation increased the risk for drug discontinuation before 24 months of therapy (*HR* = 0.6, 95% *CI* (0.35–0.99), *p* = 0.04).

**Conclusions:**

Most pediatric patients are forced to switch to either more efficacious 1st line or 2nd line drugs. Additionally, our study suggests that older age at the time of the 1st line treatment initiation, contributes to earlier drug discontinuation.

**Supplementary Information:**

The online version contains supplementary material available at 10.1007/s10072-022-06431-y.

## Introduction

Pediatric-onset multiple sclerosis (POMS) occurs in approximately 5–10% of multiple sclerosis patients (MS) with the most common disease course being the relapsing–remitting (RRMS) [1]. As in the adult-onset multiple sclerosis (AOMS), several genetic, epigenetic, and hormonal risk factors have been linked to disease development [2, 3]. Contrary to AOMS patients, POMS subjects typically display a more inflammatory-active disease course, resulting in more frequent relapses, but slower long-term disability accumulation [4]. Additionally, rapid accumulation of white and gray matter damage as well as worse long-term physical and cognitive disability are typical features of POMS [5–7]. These features are generally attributed to the extensive ability for myelin repair/synthesis and greater plasticity of the developing brain [8]. Despite the relatively slower physical disability progression, POMS more frequently display impaired brain development and poorer cognitive performance compared to AOMS, as a result of the earlier and more frequent inflammatory attacks [9, 10].

Regardless of the great experience that has been accumulated in the treatment of pediatric MS in the last decade, the optimal therapeutic option remains uncertain. The 1st line therapies (also known as platform therapies) including injectables, such as interferons and glatiramer acetate have been widely used in POMS, mainly based on data derived on retrospective and open-label studies. Moreover, the newer oral disease modifying treatments (DMTs) such as teriflunomide and dimethyl fumarate have been also established as valuable treatment options for POMS therapy [11–17].

Current treatment guidelines for POMS are based on retrospective observational data, case series, prospective safety data, and expert’s opinion. The International Pediatric Multiple Sclerosis Study Group (IPMSSG) advised that treatment decisions should be tailored to each child and taken after discussing risks and benefits with the patient and the family [18, 19].

In the current retrospective study, we present data from 27 POMS patients treated with 1st line DMTs, either injectables or oral, including interferons, glatiramer acetate, teriflunomide, and dimethyl fumarate, in order to describe effectiveness and safety. Additionally, we report the patients’ human leukocyte antigens (HLA) genotype, the “gold standard” for attributing genetic burden in both AOMS and POMS [20, 21], thus incorporating possible immunogenetic correlations to their disease course and outcome.

## Materials and methods

### Patients

In the current retrospective study, 27 POMS patients, of Hellenic origin, fulfilling the 2007 and/or 2013 IPMSSG criteria for POMS diagnosis [19] and treated with 1st line DMTs as initial therapy before the age of 19 years have been included. All patients have been followed up from the 1st of January 2010 until the 31st of December 2021, at the outpatient clinic of the Multiple Sclerosis and Demyelinating Diseases Unit (MSDDU), of the 1st Neurological Department, University Hospital “Aeginition,” of Medical School, National and Kapodistrian University of Athens (NKUA). The study received ethical approval from the Hospital Ethics Committee, and written informed consent was obtained from all patients and legal guardians, according to the Declaration of Helsinki.

A detailed clinical history was obtained, followed by thorough clinical examination. Lumbar puncture (LP), magnetic resonance imaging (MRI), and routine laboratory tests were carried out, according to the existing protocols for children and adolescents with demyelinating diseases. Anti-John Cunningham virus (JCV) antibodies’ assessment was performed in patients’ serum, at Unilabs, Copenhagen, Denmark. Cell-based assays for antibodies against aquaporin-4 (AQP4) and myelin oligodendrocyte glycoprotein (MOG) were performed at the Neuroimmunology Unit, Dept. of Pathophysiology, National and Kapodistrian University of Athens, Greece, with standard protocols.

All POMS patients underwent thorough neurological examination, including EDSS assessment every 6 months, routine hematological tests every 6 months, and brain MRI scans (including brain and spinal cord scan) in 3 Tesla magnetic field, every 6 months. Enlargement of previously existing lesions has been also evaluated. Data regarding the number of relapses, calculating the annualized relapse rate (ARR), yearly EDSS scores and EDSS progression before and after treatment with 1st line DMTs have been collected.

Relapses have been evaluated by neurologist, according to the classic definition (i.e., appearance of a neurologic deficit that lasts ≥ 24 hours in the absence of fever and infection). EDSS progression was defined as increase in EDSS score of ⩾ 1.5 points from an EDSS score of 0.0, ⩾ 1.0 point from an EDSS score of 1.0–5.5, or ⩾ 0.5 point from an EDSS score ⩾ 6.0. Patients were assessed every 24 weeks [22]. MRI activity was defined as appearance of at least 1 new/enlarging T2 lesion and/or at least 1 gadolinium-enhancing lesion. No MRI activity or elimination of MRI activity has been defined as disappearance of previous lesions.

### HLA-DRB1 genotyping

HLA genotyping was performed at the Research Immunogenetics Laboratory of the 1st Department of Neurology at “Aeginition” University Hospital. Nineteen (19) patients and legal guardians provided written informed consent regarding immunogenetic testing. High molecular weight DNA was extracted from whole peripheral blood samples (8 mL peripheral blood in sodium citrate, ACD Vacutainer tube) using the DNA extraction, Maxi Kit (QIAGEN, Germany) as per manufacturer’s instructions. HLA class II (HLA-DRB1) frequencies were determined by molecular techniques for all the specificities included in the HLA nomenclature of 2012 (we present only the first two digits of each allele, for low resolution, respectively). The HLA-DRB1 genotyping was performed using a PCR-SSO (polymerase chain reaction, sequence-specific oligonucleotide) technique as previously described [21].

### Statistical analysis

Non-parametric tests were used for all the above evaluations, due to the irregularity of the quantitative variables. Univariate comparisons were made using Mann–Whitney and chi-square tests for quantitative and categorical variables, respectively. Multivariate and Cox regression were also performed. Statistical analyses were performed using SPSS 22 (SPSS Inc., Chicago, IL, USA).

## Results

### Baseline demographics and clinical characteristics of POMS patients

In the current retrospective study, 27 POMS patients have been included. The mean age at disease onset was 14.5 ± 2.6 (mean ± SD) years. The mean disease duration was 3.4 ± 4.1 years, and the mean age of patients at the period starting the 1st line DMT treatment was 16.7 ± 3.1 years. Among these patients, 17 were females (17/27, 63%), and 10 were males (10/27 37%). All patients were diagnosed with RRMS. The main clinical baseline characteristics of the study population are summarized in Table 1.

**Table 1:** Baseline characteristics of POMS patients before receiving any 1st line disease modifying treatment

Total sample (*n* = 27)	
Female	17/27 (63%)
Age at disease onset (mean ± SD)	14.5 ± 2.6
Disease duration in years (mean ± SD)	3.4 ± 4.1
Age at 1st line therapy initiation (mean ± SD)	16.7 ± 3.1
Type of MS (*n*%)	
RRMS SPMS PPMS	27/27 (100%) 0 0
First symptom (*n*%)	
Motor Sensory Optic neuritis Diplopia Cerebellar	3/27 (11%) 11/27 (41%) 5/27 (18%) 5/27 (18%) 3/27 (11%)
OCBs (*n*%)	2/27 (7%)
Relapses before treatment (mean ± SD)	4.6 ± 3.3
EDSS score before treatment (mean ± SD)	1.8 ± 0.6
EDSS progression before treatment (mean ± SD)	1.5 ± 0.8
ARR before treatment (mean ± SD)	1.4 ± 0.7
MRI activity before treatment (*n*%)	60% (16/27)
Cervical cord lesions (*n*%)	22/27 (81%)
Thoracic cord lesions *n* (%)	19/27 (70%)
Brain atrophy (*n*%)	0/27 (0%)
Spinal atrophy (*n*%)	0/27 (0%)

*POMS:* pediatric onset multiple sclerosis; *RRMS:* relapsing–remitting multiple sclerosis; *SPMS:* secondary-progressive multiple sclerosis; *PPMS:* primary-progressive multiple sclerosis; *EDSS:* Expanded Disability Status Scale; *MRI:* magnetic resonance imaging

### Treatments

Among the 27 POMS patients included in the study, 7 patients received IFN-β-1α intramuscularly (i.m.) (Avonex®) and 9 subcutaneously (s.c.) (Rebif® or Plegridy®), while 3 patients were treated with IFN-1β (Betaferon®). Three of the newer immunomodulatory agents have been administered of label, particularly: Glatiramer acetate (Copaxone®) has been administered in 3 patients, dimethyl fumarate (Tecfidera®) in 2 and Teriflunomide (Aubagio®) in 3 patients (Table 2). The mean therapy duration for IM IFN-β-1α was 17 ± 8.0 months, for IFN-1β 48 ± 8.5, for glatiramer acetate 9.0 ± 5.0, for IFN-β-1α (S.C.) 28 ± 28, for dimethyl fumarate 21 ± 21 and for pegylated IFN-β-1α (i.m.) 36 months.

**Table 2:** First line disease modifying treatment assignment in POMS patients

Initial 1st line DMT treatment								
Active substance/commercial brand name	Full cohort	IFN-β-1α Avonex®	IFN-β-1α Rebif®	IFN-β-1β Betaferon®	IFN-β-1-α Plegridy®	Glatiramer Copaxone®	Teriflunomide Aubagio®	Dimethyl fumarate Tecfidera®
Number of patients treated	27	7	8	3	1	3	3	2
Route of administration		IM	SC	SC	SC	SC	PO	PO
Adverse events	4/27	Flu-like symptoms (3/7)					Dizizness and vomiting (1/3)	

*POMS:* pediatric onset multiple sclerosis; *DMT:* disease modifying treatment; *IM:* intramuscular; *SC:* subcutaneous; *PO:* per os

Until the final follow-up of the study, only 2 (2/27) patients, receiving dimethyl fumarate, remained on the initial treatment, while all the other (25/27) discontinued and received another drug. In our total sample until the last follow-up of the study, 9 patients had 2 drug switches; 7 patients had 3 drug switches; while 2 patients had 4 drug switches. Up today, all patients have been switched to one or more agents approved as second-line treatment, in Greece and generally in Europe. The first drug discontinuation occurred after a mean of 29.5 ± 37 months and the second after a mean of 17 ± 13 months. The first therapeutic choice after the 1st DMT discontinuation was mainly natalizumab or fingolimod (considered as 2nd line therapies in Greece), while 2 patients switched to i.m. IFN-β-1α, 2 to glatiramer acetate, 1 to s.c. IFN-β-1α, 1 to i.m. pegylated IFN-β-1α, 2 to dimethyl fumarate and 2 to teriflunomide. All patients in the second switch (3^rd^ drug administered) received a 2nd line therapy (natalizumab or fingolimod or ocrelizumab).

The main reason for drug discontinuation was the inefficacy of the 1st line therapies to eliminate the MRI scans’ activity, to reduce patient’s EDDS score, or the intolerance of flu-like symptoms and the reduced compliance with the injectables.

### Clinical outcomes

#### Comparison of clinical outcomes at baseline and at the end of first line treatment

**Fig. 1:**
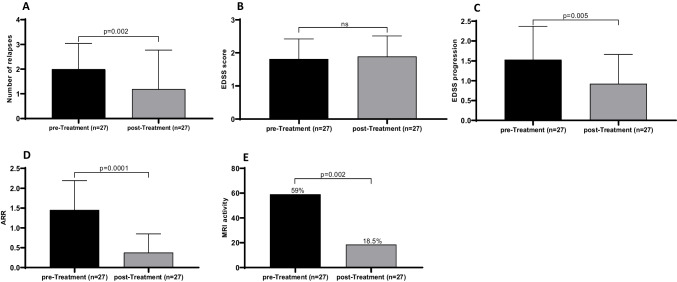
Clinical outcomes of POMS patients after treatment with 1st line DMTs. POMS: pediatric-onset multiple sclerosis; ARR: annual relapse rate; MRI: magnetic resonance imaging; EDSS: Expanded Disability Status Scale; EDDS progression: Expanded Disability Status Scale progression; DMTs: disease modifying treatments; ns: non-significant; *p* ≤ 0.05. *Pre- and post-treatment MRI activities are expressed as frequencies. All the other variables are expressed as mean ± SD

As shown in Fig. 1, after treatment with any 1st line therapy, a significant reduction in the number of relapses (mean ± SD: 2.0 ± 1.0 vs 1.2 ± 1.6, *p* = 0.002, panel A), EDSS progression (mean ± SD: 1.5 ± 0.8 vs 0.9 ± 0.7, *p* = 0.005, panel C), ARR (mean ± SD: 1.5 ± 0.7 vs 0.4 ± 0.5, *p* = 0.0001, panel D) was observed, but not in the EDSS score (mean ± SD: 1.8 ± 0.6 vs 1.9 ± 0.6, *p* = 0.60, panel B). Moreover, a significant decline-but not elimination-in the number of patients displaying MRI activity has been also observed (59% vs 18.5%, *p* = 0.002, panel E).

**Fig. 2:**
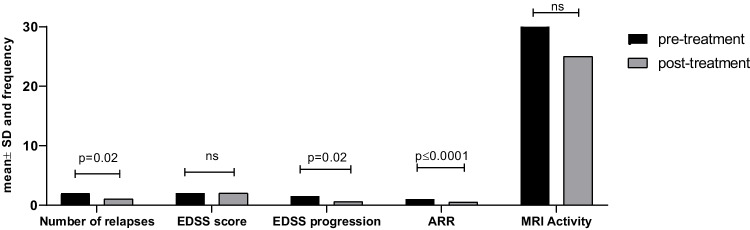
Clinical outcomes of POMS patients before and after interferon treatment. POMS: pediatric-onset multiple sclerosis; ARR: annual relapse rate; MRI: magnetic resonance imaging; EDSS: Expanded Disability Status Scale; EDDS progression: Expanded Disability Status Scale progression; ns: non-significant; *p* ≤ 0.05. *Pre- and post-treatment MRI activities are expressed as frequencies. All the other variables are expressed as mean ± SD

Furthermore, subgroup analysis revealed that patients treated with interferons (intramuscular and subcutaneous), had a significant reduction in terms of number of relapses (mean ± SD: 2.1 ± 1.2 vs 1.4 ± 1.7, *p* = 0.02), EDSS progression (mean ± SD: 1.6 ± 0.9 vs 0.9 ± 0.8, *p* = 0.02), ARR (mean ± SD: 1.5 ± 0.8 vs 0.4 ± 0.4, *p* ≤ 0.0001) post-treatment. No difference was observed in EDSS score after interferon administration (mean ± SD: 1.9 ± 0.6 vs 2.0 ± 0.6, *p* = 0.4) and MRI activity (54% vs 48%, *p* = 0.08) (Fig. 2).

Specifically, both i.m. and s.c. IFN-β-1α achieved a significant reduction in ARR compared to pre-treatment status (mean ± SD: 1.4 ± 0.5 vs 0.5 ± 0.3, *p* = 0.03 and 1.2 ± 0.5 vs 0.4 ± 0.4, *p* = 0.03) (data not shown). No other significant changes were observed in number of relapses, EDSS, EDSS progression, and MRI activity after interferon treatment or the other 1st line agents. Additionally, following further subgroup analysis, no differences in any clinical parameter were found between injectables and oral 1st line DMTs, (Supplementary Fig. 1).

#### Predictors of treatment discontinuation

**Fig. 3:**
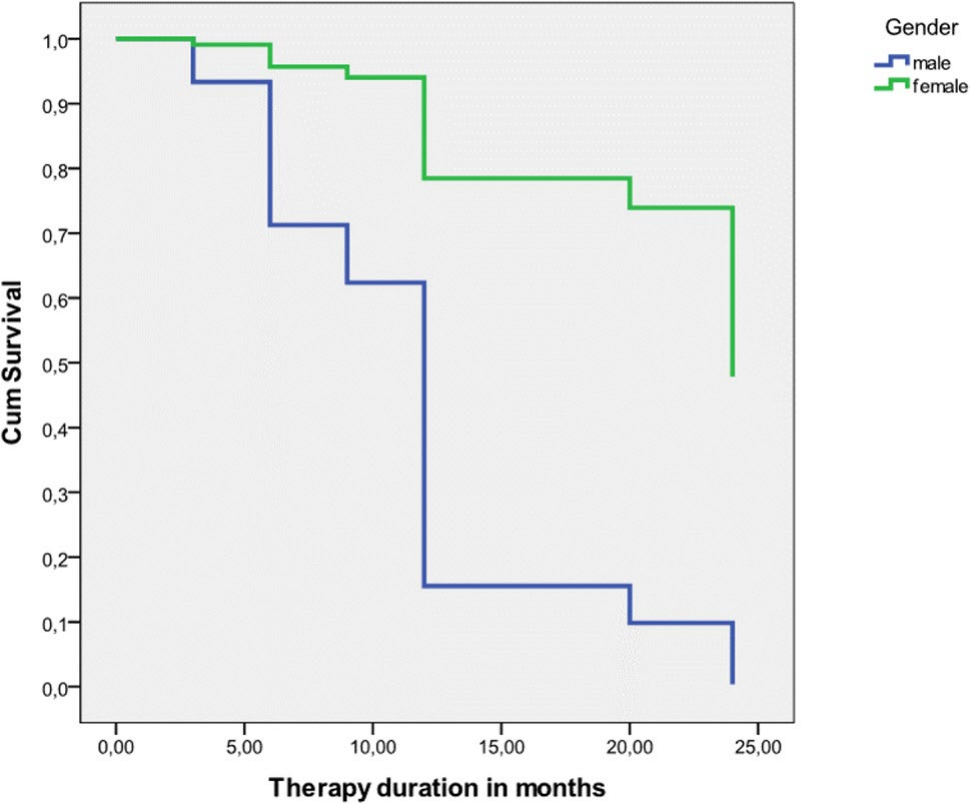
Kaplan–Meier-matched comparisons between male and female with PΟMS for the time to treatment discontinuation. POMS: pediatric-onset multiple sclerosis; Cum survival: Cumulative survival

We next sought to explore whether the time until the 1st line therapy discontinuation is associated with any demographic, laboratory, or clinical characteristic. Cox regression analysis revealed that the more advanced age of the 1st line treatment initiation, reduced the probability to continue the therapy after 24 months, for approximately 40% [Crude *HR* = 0.6, 95% *CI* (0.35–0.99), *p* = 0.04]. Moreover, a trend that males have approximately 8 times higher risk to discontinue therapy after 24 months of treatment was also observed [Crude *HR* = 7.7, *CI* 95% (0.78–78.5), *p* = 0.06] (Fig. 3).

#### Safety

Regarding safety, four patients treated with interferons have experienced flu-like syndrome. One (1) patient treated with teriflunomide had dizziness and vomiting. No adverse events (AEs) have been reported by the other 22 patients (Table 2).

#### HLA genotyping results

Ten (10) distinct HLA-DRB1 alleles were identified (number of total alleles:33) in 19 POMS patients who provided informed consent for HLA-DRB1 genotyping (Supplementary Table 1). Only one patient was homozygote for HLADRB1*16 and 18 were heterozygotes. The most common alleles were HLADRB1*11 (7/33) and HLADRB1*15 (7/33), while the less representative was HLADRB1*08 (1/33) and HLADRB1*12 (1/3). Following subgroup analysis according to the presence or not of the 4 most representative HLA-DRB1 alleles (DRB1*11, *15*, *04*, and *16*) in our sample, no association was found between any of these alleles and any post-treatment clinical outcome (data not shown).

## Discussion

This is a single-center retrospective study of 27 Hellenic POMS patients treated with 1st DMTs, including interferons, glatiramer acetate, teriflunomide, and dimethyl fumarate. The primary aim of our study was to report data regarding efficacy and safety from our POMS cohort, initially treated with any of the established 1st line therapies.

We report a good clinical response following the 1st line DMT administration. Specifically, a significant post-treatment reduction of relapse number, ARR, and EDSS progression was observed. Lower-but-not eliminated-MRI activity was also seen. Of note, eleven patients (11/27, 41%) showed a marginal EDSS score worsening during therapy. In general, our findings are in line with previous studies on POMS subjects, showing effectiveness in ARR reduction after receiving 1st line therapies [23–25].

Interferons were the most common initial treatment. Specifically, i.m. and s.c. IFN-β-1α were administered in 7 and 8 POMS patients, respectively. Both showed efficacy in ARR and EDSS progression reduction but not in the number of relapses, EDSS score, and MRI activity elimination. Similarly, previous observational studies conducted in POMS patients have reported that i.m. IFN-β-1α led to a significant reduction of the ARR, from 1.9 pre-treatment to 0.4 [26] and s.c. IFN-β-1α from 1.8 before treatment to 0.8 during the 2-year study. Of note, in both studies, no reduction in the EDSS score was observed in the majority of patients [27].

Despite the efficacy of the 1st line DMTs, most of our patients have switched to a different drug approximately after 2.5 years of treatment with the first therapy. Intramuscular IFN-β-1α was the most frequent therapy initially given, due to administration once per week. However, i.m. IFN-β-1α was also the drug which has been discontinued earlier compared to the others. In the first switch, most patients escalated to a 2nd line treatment (natalizumab or fingolimod), while in the second switch all patients escalated to a 2nd line drug (natalizumab or fingolimod or ocrelizumab). These findings are indicative of the need to treat these patients with higher potency agents. In accordance, a previous study reports that 22 of 46 patients treated with 22 mg s.c. IFN-β-1α had to escalate to higher dose (44 mg) [27]. Moreover, a recent multicenter study reported that 1/5 patients treated with a platform drug discontinued therapy approximately for 6 months due to disability worsening and were forced to switch in a second-line immunosuppressive drug including natalizumab and fingolimod [23]. In this context, a very recent retrospective study conducted in 92 Italian POMS patients showed the high efficacy of natalizumab in disease control [28], results that are in line with data published from our research group in a smaller Hellenic cohort [29, 30].

Three patients were treated with glatiramer acetate. Small cohort studies conducted in Italian POMS patients investigating the effectiveness of glatiramer acetate reported a significant reduction in relapse rate from 3.1 before treatment initiation to 0.2 during the follow-up [31, 32]. However it has been reported that highly active patients are poor responders to the glatiramer acetate [32]. Due to the small number of patients in the presented cohort, only a trend for reduction in ARR and relapse number has been observed.

Teriflunomide has been administered in 3 patients, while 2 have been treated with dimethyl fumarate. Recent published data from the TERIKIDS trial (trial number NCT02201108) support the efficacy of teriflunomide to reduce the number of new or enlarged T2 lesions by 55% and the number of gadolinium-enhancing lesions by 75%, compared to placebo. Our patients showed no elimination of MRI activity and a trend for reduction in ARR and number of relapses after teriflunomide administration, but a worsening in EDSS and EDSS progression. Additionally, a recent study reporting real-word effectiveness data suggested that initial treatment of POMS patients with newer DMTs including dimethyl-fumarate and teriflunomide led to better disease activity control, compared to injectables (interferons and glatiramer acetate), showing greater effectiveness of the newer therapies [33].

The safety and efficacy of dimethyl fumarate in POMS treatment have been recently studied in a small, single-arm, open-label trial (FOCUS study; NCT02410200) and its extension study (CONNECT study; NCT02283853). Treatment with dimethyl fumarate resulted in a significant radiological improvement as well as a reduction of the ARR from 1.5 for the year before entry into the study to 0.8 during the 24-week study period [34]. The two most frequently reported AEs were flushing (25%) and disease relapse (20%) [24]. Both patients from our cohort that have been treated with dimethyl fumarate had a reduction (not significant) in number of relapses ARR, EDSS progression, and no elimination of MRI activity. No AEs have been reported.

The main finding of our study is that POMS patients starting 1st line DMTs in more advanced age display increased risk for treatment discontinuation before 24 months of therapy, suggesting that younger patients could be better responders. This data are in line with a recent Italian retrospective study reporting that starting therapy before 12 years of age could lead to a more favorable outcome [23]. Interestingly, a trend that male POMS patients display higher risk for therapy discontinuation before 24 months of treatment, compared to females was also observed. This observation could be indicative of the reduced adherence to therapy, of male patients; however, further studies in larger cohorts should clarify this issue.

Regarding safety, all therapies were generally well tolerated, and no serious AEs have been registered. Three patients treated with i.m. IFN-β-1α showed flu-like symptoms, while 1 patient treated with teriflunomide had dizziness and vomiting. Hepatoxicity or pancreatitis, previously reported after glatiramer acetate [35] and teriflunomide [16], respectively, have not been observed in our cohort.

Regarding HLA genotyping, 7 patients were carriers of HLADRB1*15, while 2 patients carried the HLADRB1*03 allele. The predisposing role of HLADRB1*15 in POMS has been well appreciated, supporting a genetic similarity with AOMS [36], while HLA-DRB1*03 has been associated with increased disease risk in the Hellenic POMS population, as well as with higher number of relapses and lesion burden at the thoracic spinal cord level [21].

The rest of our patients carried at least one of the protective alleles HLA-DRB1*04, HLA-DRB1*08, HLA-DRB1*11, HLA-DRB1*12, HLA-DRB1*13, HLA-DRB1*14, and HLA-DRB1*16 [37]. According to our previous data, patients with disease onset ≤ 15 years showed complete absence of the HLA-DRB1*16 allele, while those with disease onset 16–19 years old presented with a lower HLA-DRB1*11 allele frequency compared both to the pediatric- and the adult-onset MS group [38].

Previous studies conducted in RRMS patients suggest that patients carrying the HLA-DRB1*04 allele and the HLA-A*03-B*44-DRB1*04 haplotype are better responders to IFN-β treatment [39], while the presence of HLA-DRB1*04:01 and HLA-DRB1*07:01 alleles has been linked to the development of interferon-neutralizing antibodies and poor response to treatment [40]. Regarding our cohort, 3 POMS patients carrying the HLA-DRB1*04 allele developed flu-like symptoms.

Our study presents several limitations including the relatively small cohort and the limited number of patients treated with the newer 1st line DMTs glatiramer acetate, teriflunomide, and dimethyl fumarate. Additionally, given that the present study is retrospective, the patients have been followed-up for different periods. Larger scale prospective studies in the Hellenic MS population are currently underway and hopefully will shed more light also on possible immunogenetic/phenotypic correlations.

In conclusion, our study supports that despite the effectiveness of the 1st line treatments in the reduction of relapse number, ARR and EDSS progression, they fail to reduce EDSS score and eliminate MRI activity. Therefore, most pediatric patients with MS are forced to switch to either more efficacious first-line or second-line drugs. Of interest, our study suggests that the more advanced patients’ age at the moment of the 1st line treatment initiation and possibly the male gender contribute to earlier drug discontinuation.

Supplementary information.

## Supplementary Information

Below is the link to the electronic supplementary material.

Supplementary file1 (DOCX 36 KB)

Supplementary file2 (DOCX 15 KB)
