# Supplementary material for: First-line disease modifying treatments in pediatric-onset multiple sclerosis in Greece: therapy initiation at more advanced age is the main cause of treatment failure, in a retrospective observational study, with a cohort from a single Multiple Sclerosis Center
Source: Neurol Sci. 2022 Oct 5;44(2):693–701. doi: 10.1007/s10072-022-06431-y (PMC9842569; doi:10.1007/s10072-022-06431-y)
Supplement: Supplementary file 1 — Supplementary file1 (DOCX 36 KB) [file 10072_2022_6431_MOESM1_ESM.docx]

**Supplementary Figure 1**. Comparison of clinical characteristics between POMS patients treated with injectable or per os 1^st^ line DMTs.

POMS: pediatric-onset multiple sclerosis patients; DMTs: disease modifying treatment; ARR: annual relapse rate; MRI: magnetic resonance imaging; EDSS: Expanded Disability Status Scale; EDDS progression: Expanded Disability Status Scale progression; ns: non-significant; p≤0.05
