# Supplementary material for: First-line disease modifying treatments in pediatric-onset multiple sclerosis in Greece: therapy initiation at more advanced age is the main cause of treatment failure, in a retrospective observational study, with a cohort from a single Multiple Sclerosis Center
Source: Neurol Sci. 2022 Oct 5;44(2):693–701. doi: 10.1007/s10072-022-06431-y (PMC9842569; doi:10.1007/s10072-022-06431-y)
Supplement: Supplementary file 2 — Supplementary file2 (DOCX 15 KB) [file 10072_2022_6431_MOESM2_ESM.docx]

**Supplementary Table 1.** *HLA-DRB1** alleles distribution in total sample, POMS patients who received 1^st^ line DMTs.

| **HLADRB1* alleles** | **Total sample**  **(N=19), N (%)** |
| --- | --- |
| HLA-DRB1 ^*^ *03* | 2 (6%) |
| HLA-DRB1 ^*^*04* | 4 (12%) |
| HLA-DRB1 ^*^*08* | 1 (3%) |
| HLA-DRB1 ^*^*10* | 2 (6%) |
| HLA-DRB1 ^*^*11* | 7 (21%) |
| HLA-DRB1 ^*^*12* | 1 (3%) |
| HLA-DRB1 ^*^*13* | 3 (9%) |
| HLA-DRB1 ^*^*14* | 2 (6%) |
| HLA-DRB1 ^*^*15* | 7 (21%) |
| HLA-DRB1 ^*^*16* | 4 (12%) |

POMS: pediatric-onset multiple sclerosis patients; DMTs: disease modifying treatment
